# Leg Edema Quantification for Heart Failure Patients via 3D Imaging

**DOI:** 10.3390/s130810584

**Published:** 2013-08-14

**Authors:** Dieter Hayn, Friedrich Fruhwald, Arthur Riedel, Markus Falgenhauer, Günter Schreier

**Affiliations:** 1 Safety and Security Department, AIT Austrian Institute of Technology, Reininghausstr. 13, Graz 8020, Austria; E-Mails: markus.falgenhauer@ait.ac.at (M.F.); guenter.schreier@ait.ac.at (G.S.); 2 Department of Cardiology, Medical University of Graz, Auenbruggerplatz 15, Graz 8036, Austria; E-Mails: friedrich.fruhwald@medunigraz.at (F.F.); arthur.riedel@stud.medunigraz.at (A.R.); 3 Ludwig Boltzmann Institute for Translational Heart Failure Research, Stiftingtalstrasse 24, Graz 8010, Austria

**Keywords:** edema, 3D imaging, eHealth, monitoring, heart failure

## Abstract

Heart failure is a common cardiac disease in elderly patients. After discharge, approximately 50% of all patients are readmitted to a hospital within six months. Recent studies show that home monitoring of heart failure patients can reduce the number of readmissions. Still, a large number of false positive alarms as well as underdiagnoses in other cases require more accurate alarm generation algorithms. New low-cost sensors for leg edema detection could be the missing link to help home monitoring to its breakthrough. We evaluated a 3D camera-based measurement setup in order to geometrically detect and quantify leg edemas. 3D images of legs were taken and geometric parameters were extracted semi-automatically from the images. Intra-subject variability for five healthy subjects was evaluated. Thereafter, correlation of 3D parameters with body weight and leg circumference was assessed during a clinical study at the Medical University of Graz. Strong correlation was found in between both reference values and instep height, while correlation in between curvature of the lower leg and references was very low. We conclude that 3D imaging might be a useful and cost-effective extension of home monitoring for heart failure patients, though further (prospective) studies are needed.

## 1. Introduction

### 1.1. Motivation

**Figure 1.**
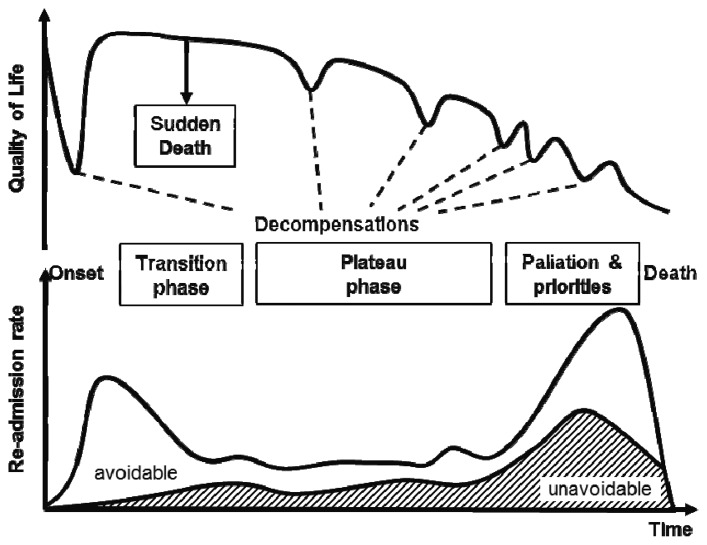
Evolution of common heart failure disease—regular rehospitalizations are common. While quality of life decreases, the necessity of care becomes more and more severe. Readmission rate increases from initial discharge to death with high ratio of avoidable readmissions in the initial phase—adapted from [5,6].

Heart failure is a common cardiac disease in elderly patients, which induces some 26,000 hospital stays each year in Austria (5% of all hospital admissions) and it is responsible for 2% of Austria's health budget [1]. After discharge, approximately 50% of all patients are re-admitted to hospital within six months [2–4]. Early symptoms of heart failure patients are often buried in noise, while the status of the patient—e.g., as measured by the NYHA class—has already deteriorated significantly. Desai *et al*. [5] showed, that especially within the initial phase after discharge (“transition phase”), most of the re-admissions to hospital could be avoided if proper treatment and monitoring was applied (see Figure 1).

Recent studies show that home monitoring of heart failure patients can reduce the number of readmissions of heart failure patients after discharge from hospital by up to 50% [7]. Still, a large number of false positive alarms (*i.e.*, low specifity) as well as underdiagnoses in other cases (*i.e.*, low sensitivity) require more accurate alarm generation algorithms, e.g., by including new kinds of sensors in existing home monitoring scenarios.

Leg edema detection could be the missing link to help home monitoring to its breakthrough: Leg edemas are a common symptom in right-sided heart failure and they are a typical sign of cardiac decompensation. Therefore, leg edema detection seems a reasonable approach to optimize home monitoring for heart failure patients.

### 1.2. State-of-the-Art

*State-of-the-art therapy of heart failure patients*: Usually, patients discharged from hospital are “left alone” with their rather complicated therapy plan, including three to five heart failure drugs in combination with additional drugs for co-morbidities. While medication is usually optimized in hospital, quite often the medication doses are inadequate for the changed settings at the patient's home, in their everyday life.

*State-of-the-art home monitoring*: Via home monitoring the evolution of the patient's status after discharge can be followed. Communication to specialists or general practitioners can be improved and in case of abnormal values, alarms can be generated and alerts can be sent to physicians/patients/ relatives *etc.* During home monitoring, different parameters are measured—usually on a daily basis. Home monitoring is usually based on body weight, blood pressure and heart rate. In some cases, medication intake, physical wellbeing or ECG are monitored as well. Medtronic has invented an implantable lung edema detection system—integrated in cardiac pacemakers or implantable cardioverter defibrillators—which is used in Europe but up to now not certified in the U.S. [8–11]. So far, no specific leg edema detection methods are used for home monitoring on a routine basis.

*State-of-the-art leg edema detection*: Leg edemas can be detected by various methods. One approach that is commonly used is continuous body weight monitoring. Fluid accumulating within the patient's legs or lungs leads to a water imbalance; less fluid is excreted than ingested and the body weight increases. Typical values are 2 kg within 2 days [12]. Unfortunately, such body weight changes can occur due to various reasons, which is why this method shows limited specifity. Additionally, no classification into lung and leg edema is possible.

Due to fluid accumulations, leg edemas change the geometry of the legs – they swell. This swelling can be measured in several ways: manual edema quantification can be done by the use of measuring tapes that are wrapped tightly around predefined positions on the leg. This method is known to show high inter-observer and even intra-observer variations.

In an attempt to measure volumes more precisely, the patient's leg can be held in a container and the amount of water displaced by the leg is measured. Since the volume of the displaced water corresponds exactly to the volume of the leg, the amount of water corresponds to the volume of the leg. Error sources of this method are described in Rabe *et al.* [13]. Additionally, a major disadvantage of this method is that it is rather complicated to apply. Trained personnel, a container of water, scales for quantifying the amount of water, *etc.* are necessary. Additionally, especially for sick patients, putting their feet into the container may be rather challenging. Even more precisely, MRI imaging could be considered an alternative, which is very precise on the one hand, but expensive and not suitable for home based monitoring on the other.

Impedance measurement is another approach for detection of edemas, which is based on the fact that the electrical properties of fluid differ from those of e.g., muscles, bones or fat. It has been used successfully for the early detection of lymphedemas in the arms [14]. By integrating the method into a cardiac pacemaker and measuring the impedance between electrode and pacemaker can, lung edemas can be detected [15]. While this method has the advantage that it is unobtrusive in case a patient already has a pacemaker, it is extremely invasive for those who don’t and this approach is suitable for lung edemas only, while it cannot be applied to leg edema detection. There is also evidence from a few publications that leg edemas can be detected by bioimpedance. One study shows significant changes in phase-sensitive single frequency impedance analysis between normal and edematous legs in patients after femoro-popliteal bypass [14]. A recent paper [16] confirms the sensitivity of single-frequency bioimpedance to leg edemas introduced by amlodipine and reports comparability to alternative methods like water displacement and circumference measurements. However, up to now this technology is not yet mature enough for application in home monitoring settings.

*State-of-the-art 3D imaging approaches for leg edema detection*: Optical 3D scanners and 3D image acquisition devices are based on various non-contact and non-intrusive depth sensing methods. Elbischger *et al.* [17] described the application of such an approach for measuring the volume of knees of patients after surgery. Kau *et al*. [18] described the use of 3D laser scanners for monitoring swellings after surgeries. Rana *et al*. [19] used optical methods for comparing different therapies against swellings with one another.

**Figure 2.**
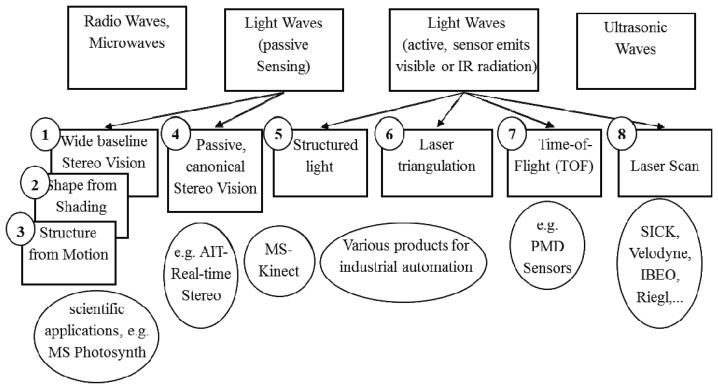
Overview of depth sensing technologies.

Figure 2 gives a brief overview of relevant methods for depth perception. Beside of a classification into active (5–8) and passive (1–4) sensors, another relevant property is whether a sensor principle requires relative motion between sensor and object in order to obtain a 3D model (2, 3, 6, sometimes 8), or if an object can be captured instantaneously by a single shot, without applying a scan-principle or the need of taking a sequence of images, e.g., under varying pre-defined patterns of structured light. This requires either quasi-static objects for the duration of 3D-measurement, or high-speed scanning or capturing capabilities. The former might induce problems related to measurement reliability and patient acceptance and the latter is reserved to high-priced sensor systems.

Methods 4, 7 and variants of 5 provide such a single-shot capability. Due to resolution limitations and relatively high costs, TOF sensors (7) appear less suitable for leg edema quantification. Thus, methods based on real-time stereo vision, structured light or a combination of those seems to be suitable for leg edema quantification. Up to now, such methods have not been evaluated for leg edema detection.

### 1.3. Aims of the Presented Work

It was the aim of the present work to analyse, whether leg edemas can be quantified in a reliable way via 3D imaging with low cost 3D image acquisition hardware.

## 2. Measurement Setup

Based on our experiences from clinical treatment of heart failure patients and from 3D imaging, we first selected geometric parameters on the leg which were expected to change during leg edemas and which can be extracted reliably from 3D images. Additionally, a sensor system for 3D image extraction was developed and algorithms for semi-automated determination of the leg edema parameters were implemented.

### 2.1. Edema Parameters

During discussions with clinical heart failure experts, two independent geometric parameters were identified, which were expected to change already in an early stage of leg edemas:

- instep height measured through three different ways
- curvature of the inner lower leg at two different heights above the inner ankle

In order to determine these parameters from 3D images of the patients’ legs, 11 reference points on the leg were defined:
A.most elevated point of the outer ankleBvertically underneath A, 1.5 cm above the surface of the scalesCcapitulum of the outer most metatarsal bone, 1.5 cm above the surface of the scalesDtip of the big toe, 1.5 cm above the surface of the scalesE.most elevated point of the inner ankleF, G, H and I1, 5, 9 and 17 cm above E along a straight line from E to the apex of the patellaJ.middle of the instep at the crossing point of lines AD and ECK.middle in between C and J

Additionally, three arbitrary points on the surface of the scales A_0_, B_0_, and C_0_ were selected.

Instep height d_1_ was defined as the distance of point J (crossing point of AD and EC) to the surface through B, C and D. d_2_ was the distance from J to the scales surface. d_3_ was defined as the distance from point K (middle of CJ) to the scales. Lower leg curvature was approximated by the distance d_4_ from H to line FI and by d_5_, the distance from G to the line FH.

**Figure 3.**
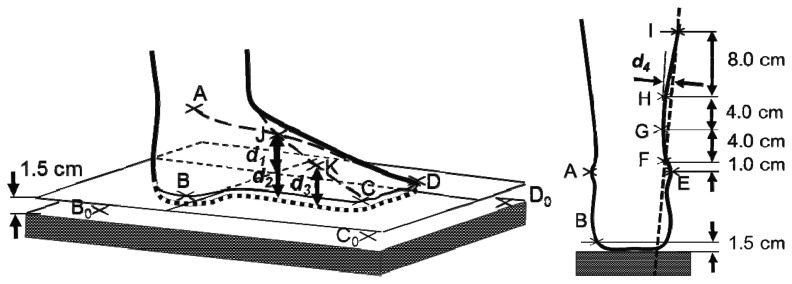
Illustration of reference points and edema parameters. Left: Definition of instep height parameters d_1_, d_2_, and d_3_. Right: Illustration of leg curvature parameter d_4_ using points F, H, and I (d_5_ was calculated similarly but using points F, G, and H).

An illustration of the reference points and distance parameters is shown in Figure 3.

### 2.2. Sensor System

**Figure 4.**
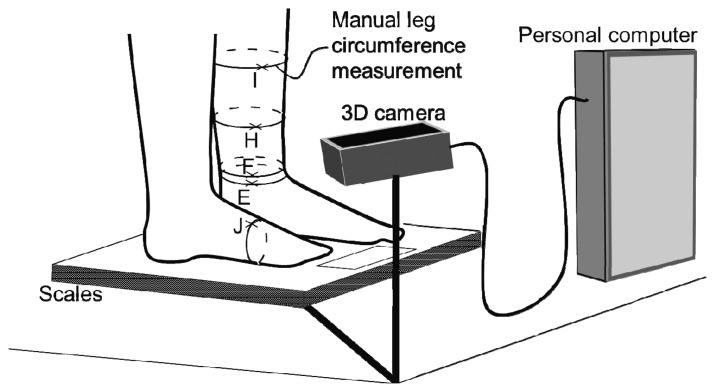
Illustration of the sensor system, consisting of a 3D camera in combination with a body weight scales, and of the five positions used for measuring the leg circumference.

A prototypical sensor system was developed based on the 3D gaming console sensor Kinect (Microsoft Corporation, Redmond, WA, USA). The Kinect is a 3D image acquisition sensor which is available for less than 100 € and for which well-developed software is freely available. By means of the Kinect's integrated colour- and depth-cameras, images of subjects standing on a body weight scales were taken. The sensor was connected to a standard personal computer that triggered image recording and stored 3D data of the subjects’ legs as well as colour information of the image. Since the geometric adjustment of camera, body weight scales and legs (standing on the scales) was fixed for all recordings, the recording depth window was constricted to the region of the scales. An illustration of the sensor system is provided in Figure 4.

### 2.3. Semi-Automated Edema Parameter Determination

Image analysis was done using Blender 2.49b (Blender Foundation, Amsterdam, The Netherlands). 3D image and colour information were overlaid and the reference points A to K were selected manually within the pictures. Starting from these points, distances d_1_ to d_5_ were calculated automatically.

Instep height d_1_ was calculated as the distance of point J to the surface BCD according to Equation (1):

(1) d1=BC→×BD→|BC→×BD→|BJ→∘

Similarly, d_2_ was calculated, again using point J, but with the surface of the scales as a reference (Equation (2)). d_2_ was expected to benefit from a more reliable definition of the reference surface, while it might be more sensitive to changes of the positioning of the foot (e.g., tilting):

(2) d2=B0C0→×B0D0→|B0C0→×B0D0→|B0J→∘

d_3_ was calculated from the surface of the scales to point K [Equation (3)]. This point close to the small toe was tested in order to evaluate whether it is more sensitive to leg edemas:

(3) d3=B0C0→×B0D0→|B0C0→×B0D0→|B0K→∘

The curvature of the inner lower legs right above the inner ankle was approximated by the distance d_4_ of point H from the line through F and I which was calculated according to Equation (2):

(4) $$\begin{array}{l} d_{4}=\frac{\left|\vec{FH}\times \vec{FI}\right|}{\left|\vec{FI}\right|}*\text{sign}(\vec{HM_{FI}}{}^{\circ}\vec{OH})\\ \text{with}\;O=\left(0,0,0\right)\;\text{and}\;M_{FI}=½\left(F+I\right) \end{array}$$

Similarly, d_5_ as a measure of the curvature closer to the ankle was calculated according to Equation (5). This distance was more related to changes of the ankle itself than to changes of the lower leg:

(5) $$\begin{array}{l} d_{5}=\frac{\left|\vec{FG}\times \vec{FH}\right|}{\left|\vec{FH}\right|}*\text{sign}(\vec{GM_{FH}}{}^{\circ}\vec{OG})\\ \mathrm{with}O=\left(0,0,0\right)\;\text{and}\;M_{FH}=½\left(F+H\right) \end{array}$$

Since 3D coordinates as provided by the Kinect were un-calibrated, all five parameters d_1_ to d_5_ were transformed to cm using the distance from the scales surface to points B, C and D (by definition 1.5 cm).

## 3. Evaluation

The system has been validated in two phases. First, the intra-subject variability of parameters d_1_ and d_4_ was analyzed when applying our method to five healthy subjects. Thereafter, correlation of the measured parameters with clinical parameters known to be related to leg edemas was calculated.

### 3.1. Evaluation of Intra Subject Variability

In spring 2012, data from five healthy subjects were analyzed in order to evaluate the stability of parameters d_1_ and d_4_ over time, when no edemas are present (parameters d_2_, d_3_ and d_5_ have been introduced after this evaluation). On day one, using a water resistant pen, optical markers were painted on the leg at the reference points described above. On subsequent days these markers have been refreshed if necessary. 3D images were taken and reference points were selected manually within the images. Instep height d_1_ and inner lower leg curvature parameter d_4_ were calculated for each measurement.

For each subject, the relative intra subject variabilities RSD (d_1_) and RSD (d_4_) were calculated as relative standard deviations according to Equation (6):

(6) $$\text{RSD}\;\left(X\right)=\frac{\text{STD}\;(X)}{MN\;(X)}*100[\%]$$

It was our assumption, that these variabilities would be much lower than changes expected during leg edemas.

### 3.2. Clinical Evaluation

During a pilot study at the Department of Cardiology of the Medical University of Graz, cardiac decompensated patients with significant leg edemas were monitored during their in-hospital stay. Each day, body weight was documented and the leg circumference was measured at five positions of the leg using a measuring tape (horizontally through point E, F, H, and I, vertically through point J, see Figure 4). Additionally, a 3D image of the legs was taken on each day and the parameters as described above were determined. The study was approved by the ethics committee of the Medical University of Graz, EK-number 24-536 ex 11/12. Furthermore, the study was registered at clinicaltrials.gov (NCT01700023).

## 4. Results and Discussion

### 4.1. Intra Subject Variations for Healthy Subjects

A total of 87 measurements at different daytimes within four consecutive days were performed for five healthy male subjects in the age range of 24 to 35 years. Manual image preparation and marker selection took less than 15 min per image. Intra subject variability for *d_1_* and *d_4_* for each single subject are summarized in Table 1.

### 4.2. Clinical Evaluation

3D images, body weight and leg circumference from 16 consecutive patients with leg edemas were recorded. Three patients were excluded since data from only one single day could be recorded and, therefore, no evolution of edema parameters could be analyzed. The remaining 13 patients (five f, age 73 ± 15 y, M ± SD) were monitored for 5.1 ± 1.9 days (M ± SD). During this period, body weight was reduced by 2.6 ± 4.9 kg (M ± SD). Details are shown in Table 2.

**Figure 5.**
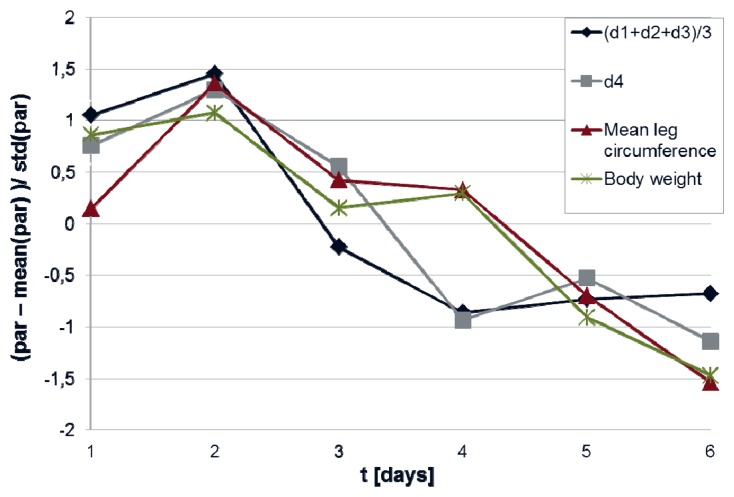
Evolution of mean instep height (d_1_ + d_2_ + d_3_)/3 and leg curvature parameter d_4_ over time for patient 2 as compared to mean leg circumference and body weight. To increase comparability, parameters were z-transformed according to z = (x − MN)/STD before plotting.

Figure 5 gives an example of the evolution of body weight and mean leg circumference as compared to the evolution of mean instep height and leg curvature.

For all patients, changes of parameters d_1_−d_5_ were correlated with changes of body weight and leg circumference. Results are shown in Table 3.

Two patients (patient 6 and patient 10) could not rise from the sitting position during data acquisition. This led to unreliable body weight values as well as unreliable edema parameters, especially concerning instep height. Therefore, all analyses were also done excluding these two patients, respectively, with the data shown in Table 4.

**Figure 6.**
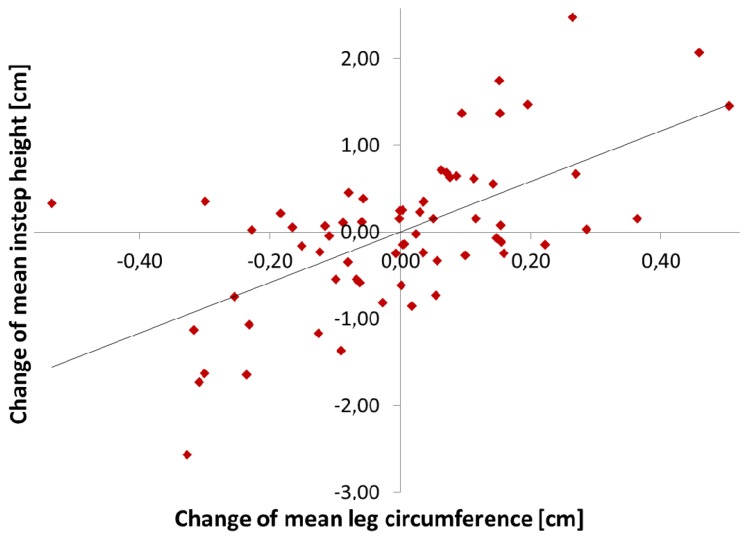
Correlation in between changes (as compared to each patient's mean value) of the mean leg circumference and changes of the mean instep height (d_1_ + d_2_ + d_3_)/3 of all but the two sitting patients (*r* = 0.63).

Correlation of mean instep height (d_1_ + d_2_ + d_3_)/3 with all reference values was high (correlation coefficient *r* > 0.60). The highest correlation was found in between mean instep height and the combined reference value (*r* = 0.64), which was slightly better than correlation with leg circumference only (*r* = 0.63). No correlation in between d_5_ (the curvature of the foot 1–8 cm above the ankles) and neither body weight nor leg circumference could be found. The curvature parameter d_4_ (1–17 cm above the ankles) showed medium correlation with leg circumference (*r* = 0.42) but low correlation with body weight (*r* = 0.18). Figure 6 plots mean instep height changes over mean leg circumference changes.

### 4.3. Suggested Implementation for Integration in a Telemonitoring Setup

Up to now, parameter extraction from 3D images was done in a semi-automated way. Especially, methods for automated detection of the reference points as defined in Section 2 are still needed. From our approach with independent sensors—*i.e.*, body weight scales and a 3D camera—new methods for analysis these additional data need to be developed, in order to decide, whether an edema is present or not. It is important to notice, that edema detection from absolute values is unlikely to be working appropriately, since leg geometry is highly depending on the individual patient. Nevertheless, changes in leg geometry can be correlated with leg edemas—especially when combined with the body weight. Table 5 summarizes possible changes in weight and leg geometry, as well as suggested reactions as required by the home monitoring system. Nevertheless, reliability of such alarms is intended to be validated in future studies.

With the proposed concept, no additional measurement device would need to be provided to the patient, since the leg edema detector can be added to the weight scales in an unobtrusive way. Furthermore, extra buttons or other user interfaces and additional user interactions would not be required, since the camera could be triggered by the body weight scales, *i.e.*, the photo could be taken at the moment the scales identifies a steady-state (which is already done in order to trigger the measurement of the body weight).

The equipment that we used required a minimum distance from the camera to the subject of approximately 70 cm. Therefore, in order to integrate the camera in a body weight scales, the optical system of the camera needs to be adapted, so that the minimum distance can be reduced to 10 cm, e.g., by the use of a commercially available play range reduction lens (Zoom, Nyko, Los Angeles, CA, USA).

There are several interesting points on the patient's leg that should be included in the image, such as inner and outer ankle, toes and instep. Therefore, it is essential that the camera is positioned in a way that all these points can be seen on the image. Since moving elements are to be avoided, the optimal position of the camera is likely to be one of the front corners (on the toe-side of the foot), elevated high enough in order to see the upper side of the instep, since all relevant points can be seen from that perspective—either on one or on the other leg.

Regular cameras in patient homes—especially in bathrooms—face severe concerns regarding privacy and user acceptance. Therefore, it is essential, that no pictures may be stored, transmitted, or displayed and that terms such as “camera”, “picture” or “photo” be avoided. Lenses and optical elements should be hidden to the user and the field of vision should mechanically be limited as much as possible. Additionally, due to the 3D camera, it is possible to restrict the region which can be seen by the camera to a 3D square which can be defined right upon the body weight scales with a height of no more than 50 cm. By that, it can be assured that nothing but the patients legs can be seen by the sensor.

### 4.4. Integration in a State-of-the-art Home Monitoring System for Heart Failure Patients

State-of-the-art home-monitoring equipment for heart failure patients already provides body weight scales with intuitive and secure data transmission to home monitoring centers [20]. Using a web-interface, authorized physicians have access to their patients’ data and notifications can be sent in case of events, such as edema alarms. Our 3D imaging based edema detector has been designed in a way that it could be integrated in such a state-of-the-art home-monitoring system, simply by exchanging standard body weight scales by new devices, which is expected to keep costs for the equipment at a manageable prize.

### 4.5. Limitations

Our results show that geometric parameters from 3D images correlate with leg circumference and body weight during leg edema reduction. Although correlation coefficients >0.60 especially for mean instep height indicate high correlation, there are some limitations.

Intra-subject variability for d_1_ and d_4_ as determined for healthy subjects was half the variability we found for heart failure patients with edemas. This means that up to now results can be reproduced with limited reliability only. As can be seen from Table 3 and Table 4, averaging of three different instep height parameters improved the results—this might be due to reduction of measurement errors. Based on the data we achieved during our study, we expect that the definition of the edema parameter d_1_-d_5_ could be improved, leading to lower intra-subject variabilities and higher sensitivity to edemas, e.g., by selecting other reference points, selecting other distances, or by special image processing approaches such as fitting individual foot models into each 3D image.

Our approach is limited to intra-subject variabilities of edema parameters—no absolut edema measure can be determined. Since only changes can be detected, it is suitable for edemas with varying amount (such as cardiac edemas) only, while it is insensitive to other edema related pathologies (e.g., varicosis), which lead to no or only slow edema changes. Although we suggested a method for implementing an edema alarm algorithm based on body weight and intra-subject variability of 3D imaging parameters, reliability of such an algorithm has not been validated yet.

One problem we identified is the reliability of the reference values which were used as a golden standard. As described in the introduction, our first reference value—body weight—depends on several influences, such as nutrition and lung edemas, e.g., one of our patients

Additionally, during the study we found that leg circumference strongly depends on: (a) behavior of the patient right before measurement (e.g., lying in bed *vs.* walking) and (b) on the socks worn. Especially surgical stocking, as often used during treatment of leg edemas, has a great influence on the reference data. In a real-life scenario, several other factors are expected to influence the results (e.g., water consumption, excretory system, alcohol consumption, drugs used—especially diurectics, time of measurement, *etc.*). Additionally, intra-subject variability of healthy subjects was studies for male subjects only, while heart failure patients were both male and female, which might have affected the results. Up to now, it is not known, which parameter are affected by these aspects. Nevertheless, currently leg edemas are mostly detected using the body weight only. Therefore we expect, that by introducing an independent new measure based on 3D imaging, reliability of edema detection could be improved.

We expect that leg circumference measured at five positions of the foot is closely related to leg volume. Nevertheless, state-of-the-art foot volume measurement is usually done via water displacement. This method might be slightly more precise, but it has the disadvantage that measurements would become much more complicated. Even using normal scales, two of our patients could not be measured in a standardized way. Making patients step into a water container would probably have led to even more patients that need to be excluded.

Even leg volume itself may not be the optimal reference value for evaluating sensors for leg edemas. As during our study, foot clothing and physical activities influence the leg volume, while the amount of fluid within the legs might stay constant (though distributed differently in between upper and lower regions of the leg). From a pragmatic point of view, neither body weight nor leg volume should be the golden standard, but the overall physiological status of the patient. Most importantly, cardiac decompensations should be detected. Up to now it is not known, which parameter (leg volume, body weight, *etc.*) is most sensitive and specific in this aspect.

## 5. Conclusions/Outlook

We conclude that 3D imaging might be a useful and cost-effective extension of home monitoring for heart failure patients. Three of our leg edema parameters—those quantifying the instep height (d_1_, d_2_ and d_3_)—were strongly correlated with other leg edema related parameters, *i.e.*, body weight and leg circumference. Combining such parameters with state-of-the-art body weight measurements has the potential to significantly improve alarm management in home monitoring scenarios in terms of sensitivity and specifity optimization.

Following the present results, the next step will be to implement the concept illustrated in the present paper, and to test the approach in a real life scenario. Thereafter we hope to know, whether 3D imaging can improve sensitivity and specifity of alarm generation algorithms and—as the final aim—to detect cardiac problems earlier than what is possible with existing systems and to further improve patient management by home monitoring of heart failure patients.

## Figures and Tables

**Table 1.** Intra-subject variability of instep height d_1_ and inner lower leg curvature approximation d_4_ for all healthy subjects.

**Subject**	**Sex**	**Age [y]**	**Monitoring Period [d]**	**n Measurements [1]**	**RSD(d_1_) [%]**	**RSD(d_4_) [%]**
Healthy subject 1	m	27	4	15	1.51	9.43
Healthy subject 2	m	35	4	21	2.94	7.65
Healthy subject 3	m	27	4	21	2.62	7.54
Healthy subject 4	m	24	4	21	3.53	8.43
Healthy subject 5	m	29	4	21	3.81	8.55
**Mean**	n.a.	**28.4**	**4**	**19.8**	**2.89**	**8.32**
**Standard Deviation**	n.a.	**4.1**	**0**	**2.7**	**0.90**	**0.77**

**Table 2.** Basic data of the study population.

**Patient ID**	**Sex**	**Age** **[y]**	**Monitoring Period** **[d]**	**Body Weight [kg]**			**Edema Location**	
	
				**first day**	**last day**	**difference**	**leg**	**foot**
1	f	76	6	85.6	84.0	−1.6	yes	yes
2	m	37	6	112.3	109.0	−3.3	yes	yes
3	f	83	4	68.0	66.4	−1.6	yes	yes
4	m	77	4	75.9	74.8	−1.1	marginal	yes
5	f	85	4	56.6	56.7	0.1	marginal	yes
6 (sitting)	f	93	3	52.4	60.0	7.6	yes	yes
7	f	89	4	86.4	83.4	−3.0	yes	yes
8	m	59	6	105.7	98.9	−6.8	yes	yes
9	m	84	7	95.4	88.0	−7.4	yes	yes
10 (sitting)	m	70	4	66.9	66.8	−0.1	yes	yes
11	m	78	4	81.1	80.0	−1.1	yes	yes
12	m	71	10	100.8	87.2	−13.6	yes	yes
13	m	67	4	112.4	110.1	−2.3	yes	yes
**Mean**	n.a.	**73**	**5.1**	**84.6**	**81.9**	−**2.6**	**n.a.**	**n.a.**
**Std**	n.a.	**15**	**1.9**	**20.1**	**17.1**	**4.9**	**n.a.**	**n.a.**

**Table 3.** Correlation in between changes of 3D parameters (as compared to each patient's mean value) and changes of leg circumference and body weight (strong correlation > 0.6 is plotted in **bold**, low correlation < 0.4 in *italic*).

	**Body Weight**	**Leg Circumference**	**Mean (References)**
d_1_	0.50	**0.61**	0.56
d_2_	0.46	0.53	0.50
d_3_	0.56	0.57	0.59
instep_mean_	0.55	**0.63**	**0.60**
d_4_	−*0.01*	*0.26*	*0.08*
d_5_	−*0.12*	−*0.10*	−*0.12*

**Table 4.** Correlation in between changes of 3D parameters (as compared to each patient's mean value) and changes of leg circumference and body weight after exclusion of sitting patients (strong correlation > 0.6 is plotted in **bold**, low correlation < 0.4 in *italic*).

	**Body Weight**	**Leg Circumference**	**Mean (References)**
d_1_	0.54	**0.61**	0.58
d_2_	0.53	0.54	0.55
d_3_	**0.61**	0.59	**0.63**
instep_mean_	**0.60**	**0.63**	**0.64**
d_4_	*0.22*	0.42	*0.29*
d_5_	−*0.19*	−*0.11*	−*0.17*

**Table 5.** Interpretation of leg geometry based on 3D imaging and body weight for edema detection.

		**Leg Volume**		

		**Increasing**	**Constant**	**Decreasing**
**Body Weight**	Increasing	**Leg edema alarm**	Borderline	No leg edema alarm *(lung edema? nutrition?)*
	Constant	Borderline	No leg edema alarm	No leg edema alarm
	Decreasing	No leg edema alarm *(leg injury?)*	No leg edema alarm	No leg edema alarm
